# miR-223 regulates migration and invasion by targeting Artemin in human esophageal carcinoma

**DOI:** 10.1186/1423-0127-18-24

**Published:** 2011-03-31

**Authors:** Shujun Li, Zhigang Li, Fengjie Guo, Xuebo Qin, Bin Liu, Zhe Lei, Zuoqing Song, Liya Sun, Hong-Tao Zhang, Jiacong You, Qinghua Zhou

**Affiliations:** 1 Tianjin Key Laboratory of Lung Cancer Metastasis and Tumor Microenvironment, Tianjin Lung Cancer Institute, Tianjin Medical University General Hospital, Tianjin, 300054 PR China; 2 Soochow University Laboratory of Cancer Molecular Genetics, School of Basic Medicine and Biological Sciences, Medical College of Soochow University, Suzhou, People's Republic of China

**Keywords:** miR-223, ARTN, esophageal carcinoma, migration and invasion

## Abstract

**Background:**

Artemin (ARTN) is a neurotrophic factor belonging to the glial cell-derived neurotrophic factor family of ligands. To develop potential therapy targeting ARTN, we studied the roles of miR-223 in the migration and invasion of human esophageal carcinoma.

**Methods:**

ARTN expression levels were detected in esophageal carcinoma cell lines KYSE-150, KYSE-510, EC-9706, TE13, esophageal cancer tissues and paired non-cancerous tissues by Western blot. Artemin siRNA expression vectors were constructed to knockdown of artemin expression mitigated migration and invasiveness in KYSE150 cells. Monolayer wound healing assay and Transwell invasion assay were applied to observe cancer cell migration and invasion. The relative levels of expression were quantified by real-time quantitative PCR.

**Results:**

ARTN expression levels were higher in esophageal carcinoma tissue than in the adjacent tissue and was differentially expressed in various esophageal carcinoma cell lines. ARTN mRNA contains a binding site for miR-223 in the 3'UTR. Co-transfection of a mir-223 expression vector with pMIR-ARTN led to the reduced activity of luciferase in a dual-luciferase reporter gene assay, suggesting that ARTN is a target gene of miR-223. Overexpression of miR-223 decreased expression of ARTN in KYSE150 cells while silencing miR-223 increased expression of ARTN in EC9706 cells. Furthermore, overexpression of miR-223 in KYSE150 cells decreased cell migration and invasion. Silencing of miR-223 in EC9706 cells increased cell migration and invasiveness.

**Conclusions:**

These results reveal that ARTN, a known tumor metastasis-related gene, is a direct target of miR-223 and that miR-223 may have a tumor suppressor function in esophageal carcinoma and could be used in anticancer therapies.

## Background

Artemin (ARTN) is a neurotrophic factor belonging to the glial cell-derived neurotrophic factor (GDNF) family of ligands (GFLs) [1-4], which is important in tumor growth, migration, adhesion and invasion [5,6]. Increased expression of ARTN has been identified in several human cancers including breast cancer, pancreatic cancer, thyroid carcinoma and endometrial carcinoma [5-10]. Increasing evidence had demonstrated a connection between high expression of ARTN and tumor relapse, metastasis and poor prognosis [7,8].

MicroRNAs (miRNAs) are a group of small non-coding RNAs (approximately 21-25 nt) that negatively regulate gene expression by imprecisely binding to complementary sequences in the 3' untranslated region (UTR) of their target mRNAs [11,12]. It has been confirmed that miRNA abnormalities play an important role in gene regulation, apoptosis, the maintenance of cell differentiation and tumorigenesis [13-15]. A recent study has shown that differential expression of miRNAs was correlated with esophageal carcinoma survival [16,17]. Furthermore, down-regulation of miR-223 was associated with poor prognosis in chronic lymphocytic leukemia [18,19]. There is emerging evidence that suggests that miR-223 plays an important role in cell proliferation, hematopoietic development and differentiation [20-22]. In this study, we discovered that the expression of ARTN in esophageal carcinoma tissue was higher than that of adjacent tissues, and down-regulation of artemin expression mitigated KYSE150 cell migration and invasiveness. Furthermore, ARTN is a target gene of miR-223. Regulation of miR-223 expression affected the expression of ARTN as well as cell migration and invasion in esophageal carcinoma cells. Finally, we validated that ARTN is a direct target of miR-223 in the context of human esophageal carcinoma.

## Methods

### Cell culture

The human esophageal carcinoma cell lines KYSE-150, KYSE-510, EC-9706 and TE13 were cultured in RPMI-1640 (Invitrogen, Carlsbad, CA) medium supplemented with 10% fetal bovine serum (FBS, GIBCO), 100 IU/ml penicillin and 100 mg/ml streptomycin in humidified 5% CO_2 _at 37°C. Human embryonic kidney 293 (HEK293) cells were cultured in Dulbecco's minimum essential medium (DMEM) supplemented with 10% fetal bovine serum (FBS), 2 mM L-glutamine, and 1 mM sodium pyruvate. For transfection, cells were grown to 80% confluency and transfected with RNAi vector, recombined eukaryotic vector, a chemically synthesized miRNA-223 inhibitor or a negative control using Lipofectamine 2000 (Invitrogen, CA, USA), according to the manufacturer's recommendation.

### Collection of tissues

Three samples of esophageal cancer tissues and paired non-cancerous tissues (5 cm away from tumor) were obtained from The Second Hospital of He Bei Medical University. The tissue samples were collected with written consent from the patients. The resected tissue samples were immediately cut into small pieces and snap frozen in liquid nitrogen until use. All tumor tissue and paired non-cancerous tissue samples were pathologically confirmed.

### Western blot analysis

Purified esophageal cancer antigen in cold lysate buffer (50 mmol/L Tris-Cl pH 8.0, 150 mmol/L NaCl, 10 mmol/L Triton X-100, 10 mmol/L PMSF) was separated on 10% gradient SDS-polyacrylamide gels in the presence of β-mercaptoethanol. The proteins were transferred onto a NC membrane, blocked with 50 mg/mL fat-free milk in 100 ml PBS for 2 h and incubated with the primary antibody (anti-artemin, R&D Systems) overnight at 4°C. The membrane was washed and then incubated with HRP-conjugated goat anti-rat IgG/IgM (Sigma-Aldrich) at RT for 1 h. The membrane was washed again, and the antigen-antibody reaction was visualized using an ECL detection system. The band intensity was quantified by arithmetic analysis using the software Scion Image beta 4.03. The ratio of artemin/β-actin in each sample was used for statistical analysis.

### Construction of artemin siRNA expression vectors

To knock down artemin expression, we used a pSilencer 2.1 vector encoding a small hairpin RNA directed against the target gene in KYSE-150 cells. The target sequence for artemin was 5'- AACAGCACCTGGAGAACCGTG -3' (KYSE-150/RNAi). As a negative control, we used an shRNA vector without the hairpin oligonucleotides (KYSE-150/NC).

### Monolayer wound healing assay

Migration ability was determined using a wound-healing assay. Cells were grown in 10% FBS medium on 60 mm plates. After the cells reached sub-confluence, the cells were wounded by scraping the monolayer and grown in medium for 48 h. The width of the wound was measured at different time points. Three to four different locations were visualized and photographed under a phase-contrast inverted microscope (40× objective, Leica, Solms, Germany).

### Transwell invasion assay

Cell invasion assays were performed using 24-well transwells (8 mm pore size, Corning Life Sciences) coated with matrigel (1 mg/ml, BD Sciences). Cells (10^4^/well) were seeded in the upper chambers of the wells in 200 μl FBS-free medium, and the lower chambers were filled with 500 μl 10% FBS medium to induce cell migration. Following incubation for 24 h, the cells on the filter surface were fixed with 4% formaldehyde, stained with 0.5% crystal violet, and examined under a microscope. Cells in at least six random microscopic fields (200×) were counted.

### Plasmid construction and luciferase reporter assay

The eukaryotic expression vector pcDNA3.1 (+) (Invitrogen) was used to construct the miRNA expression plasmid. The genomic sequences, including 200 bp flanking sequences, of the human miR-105, miR-223 and miR-760 genes were cloned from HEK293 cells. The PCR products were digested by *EcoR I *and *BamH I *and subcloned into the pcDNA3.1 (+) vector. The full-length 3' untranslated region (3'UTR) of ARTN was amplified from a human cDNA library; the amplified product (464 bp) was subcloned into the pMIR-GLO™ luciferase vector (pMIR, Invitrogen) downstream of the firefly luciferase coding region. The recombined vector was named pMIR-ARTN. Mutations of miR-223 binding sites were introduced by site-directed mutagenesis; four nucleotides within the core binding sites of *ARTN *3'UTR were changed. The resulting vector was called pMIR-ARTN-Mut. Primer sequences used in construction of the vectors are listed in Table 1. The sequences of the resulting reporter vectors were verified by sequence analysis.

**Table 1:** Primer sequences of expression vectors construction

Gene name	Primer name	primer sequence
miR-223	sense primer	5'-TGGATCCGTGTCACTCGGGCTTTACCTG-3'
	antisense primer	5'- CGAATTCGTAGACACAGCCCAGGGCTGT-3'
miR-105	sense primer	5'-TGGATCCGTGCTTATGCCCTTTAGCTATG-3'
	antisense primer	5'- TGAATTCCTGATGGTGCCATGCTTCCTCATATG-3'
mir-760	sense primer	5'-TGGATCCGAGCGCGCGCCCTCCGACCAC-3'
	antisense primer	5'-TGAATTCCCGTTAAGCCGGGCCGGTGAC-3'
ARTN-3'UTR	sense primer	5'- TGAGCTCGGGCTCGCTCCAGGGCTTTGCAGAC-3'
	antisense primer	5'- TCTCGAGATAGGGGCCAGCTCCCATGAGTG-3'
ARTN-3'UTR-Mut	sense primer	5'-AAGACTCTAGCAGCCCCAGAGCCCTCAC-3'
	antisense primer	5'-GTCCCTTCACCTGTTCGGGGATGA-3'

Luciferase assays were conducted using 1 × 10^4 ^HEK 293 cells plated on a 96-well plate. Co-transfection was performed using 2 ng pMIR-ARTN, pMIR-ARTN-Mut or pMIR-GLO™ empty vector and either 80 ng miRNA expression vector or pcDNA 3.1(+) empty vector. Forty-eight hours after transfection, the cells were harvested and assayed for both firefly and renilla luciferase using the dual-luciferase glow assay (Promega, Madison, WI). All transfection experiments were conducted in triplicate.

### Real-time quantitative PCR

Quantitative RT-PCR was carried out using SYBR Premix Ex Taq™ (Code DRR041A, Takara). Total RNA isolated using the mirVana Kit (Applied Biosystems, CA) was subsequently reverse transcribed to cDNA using the stem-loop reverse transcription primer for miRNA detection. Reverse transcription of ARTN mRNA was performed using M-MLV reverse transcriptase (Epicentre, Paris, France) and a random primer. The *U6 *small nuclear RNA and *GAPDH *mRNA were used as internal controls for miRNA and *ARTN *mRNA, respectively. All primer sequences are listed in Table 2. The reactions were placed in a 96-well plate (ABI) using a preheated real-time instrument (ABI 7500 HT). The relative levels of expression were quantified and analyzed using Bio-Rad iCycler iQ software. Ct values were used to calculate the levels of RNA expression. The amount of target gene expression (2^-ΔΔCt^) was normalized using the endogenous GAPDH or U6 reference; the amount of target gene in the control sample was set at 1.0.

**Table 2:** Primer sequences of products expression

Gene name	Primer name	primer sequence
U6	RT primer	5'-GTCGTATCCAGTGCAGGGTCCGAGGTATTCGCACTGGATACGACAAAATATGGAAC-3'
	sense primer	5'-TGC GGGTGCTCGCTTCGGCAGC-3'
	antisense primer	5'-CAGTGCAGGGTCCGAGGT-3'
GAPDH	RT primer	radom primer
	sense primer	5'-TGGGTGTGAACCACGAGAA-3'
	antisense primer	5'-GGCATGGACTGTGGTCATGA-3'
miR-223	RT primer	5'-GTCGTATCCAGTGCAGGGTCCGAGGTATTCGCACTGGATACGACTGGGGT-3'
	sense primer	5'-CGTTGTCAGTTTGTCAAATAC-3'
	antisense primer	5'-CAGTGCAGGGTCCGAGGT-3'
ARTN	RT primer	radom primer
	sense primer	5'-TGCTGAGCAGCGTCGCAGAG-3'
	antisense primer	5'-GCTCTTCCACTGCACCAGCG-3'

### MTT assay

Cells (1.0 × 10^4 ^cells/ml) were cultured in 96-well plates for varying periods of time and exposed to fresh media every other day. During the last 4 h of each day of culture, the cells were treated with methyl thiazolyl tetrazolium (MTT, 50 μg per well, Sigma, USA). The generated formazan was dissolved in DMSO, and the absorbance at 570 nm was measured to measure cell viability.

### Statistical analysis

Data are expressed as mean ± SEM. The difference among groups was determined by ANOVA analysis and comparison between two groups was analyzed by the Student's t-test using GraphPad Prism software version 4.0 (GraphPad Software, Inc., San Diego, CA). A value of P < 0.05 was considered statistically significant.

## Results

### Expression of ARTN in human esophageal carcinoma tissues and cell lines

**Figure 1:**
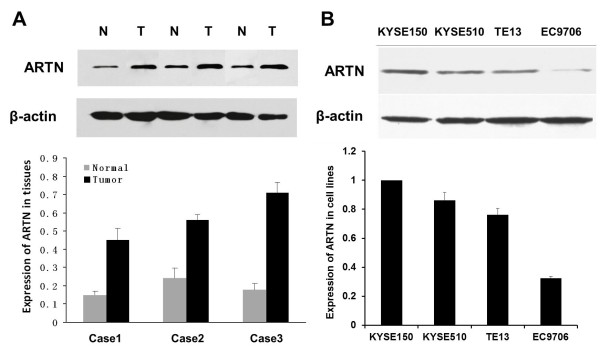
**ARTN expression in esophageal carcinoma tissues and cells**. (A) ARTN expression in esophageal carcinoma and normal tissues using Western blot (upper panel). Bar graph of the relative expression of ARTN in tissues relative to control β-actin (lower panel). (B) ARTN expression in esophageal carcinoma cells (KYSE150, KYSE510, TE13 and EC9706) (upper panel). Bar graph of the relative expression of ARTN in cells; ARTN(KYSE150) was set as control (lower panel). Data are representative of each group or expressed as mean ± SEM from three separate experiments.

To explore the role of ARTN in esophageal cancer, the expression level of ARTN in human esophageal carcinoma and normal tissues was detected by Western blot and quantified by densitometry using β-actin as a loading control. In most cases, ARTN levels in normal esophageal tissue were significantly lower than those in esophageal cancer tissue (Figure 1A). The expression level of ARTN in four human esophageal carcinoma cell lines was also examined by Western blot. As seen in Figure 1B, all four carcinoma cell lines expressed ARTN. The highest expression was detected in KYSE150 cells, while moderate expression was observed in the KYSE510 and TE13 cell lines, and low expression was observed in EC9706 cells.

### Effect of down-regulated artemin expression on the migration of KYSE150 cells

**Figure 2:**
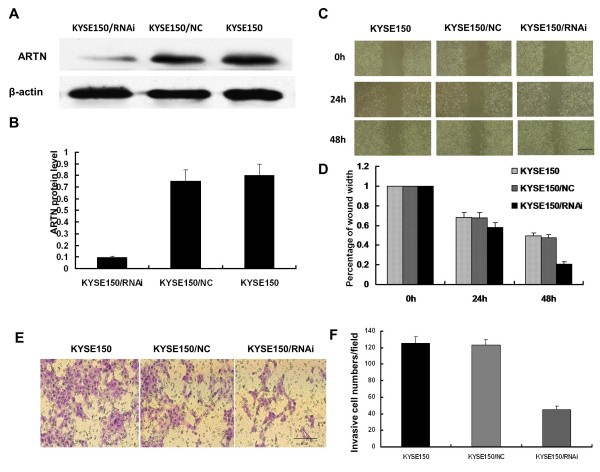
**Effect of down-regulation of ARTN on the migration of KYSE150 cells**. (A) Western blotting confirmed the knockdown of artemin expression in KYSE150 cells. (B) Bar graph of the relative expression of artemin. (C) KYSE150 cells after wounding and during healing. (D) Measurement of migration distance. (E) The filters were stained with crystal violet and inspected under a microscope. (F) Quantitative measurement of invaded cells. Scale bars in microscope is 100 μm. Data are representative of each group or expressed as mean ± SEM from three separate experiments.

Overexpression of an oncogene is crucial for the development of tumors as it can promote strong invasion of tumor cells. To determine the impact of artemin expression on the growth of KYSE150 cells, we constructed siRNA expression vectors (KYSE150/RNAi) specific to artemin transcripts and transfected them into KYSE150 cells. The knockdown was confirmed by western blot; KYSE150/RNAi was effective compared to the negative control (KYSE150/NC) and the parental KYSE150 cells [Figure 2(A, B)]. The successful knockdown of the artemin gene in KYSE150 cells provided a useful tool for investigating the function of ARTN in the growth of KYSE150 cells.

Next, we examined the impact of artemin expression on the migration of KYSE150 cells by a wound healing assay, shown in Figure 2(C). Following incubation of physically-wounded cells for 48 h, KYSE150/RNAi cells had traveled a significantly shorter distance than control cells [Figure 2(D)]. To analyze invasiveness, another important feature of malignant cells, we performed transwell invasion assays using cell culture inserts covered with extracellular matrix components. KYSE150 and KYSE150/NC cells had strong invasive abilities while inhibition of artemin resulted in a massive reduction in invasion (Figure 2E and 2F). The wound healing and invasion assays indicate that down-regulation of artemin expression reduces the migration of KYSE150 cells.

### miR-223 interacts with the ARTN 3'UTR and regulates endogenous ARTN protein expression

To identify potential miRNAs that specifically target and regulate ARTN, we used bioinformatic web-based servers including miRBase, TargetScan and Pictar, to scan the 3'UTR of ARTN for miRNAs binding sites. As a result, the three miRNAs with the highest free energy (hsa-mir-105, hsa-mir-223 and hsa-mir-760) were chosen. The seed regions for the three miRNAs for the ARTN 3'UTR are shown in Additional file 1, Figure S1A.

**Figure 3:**
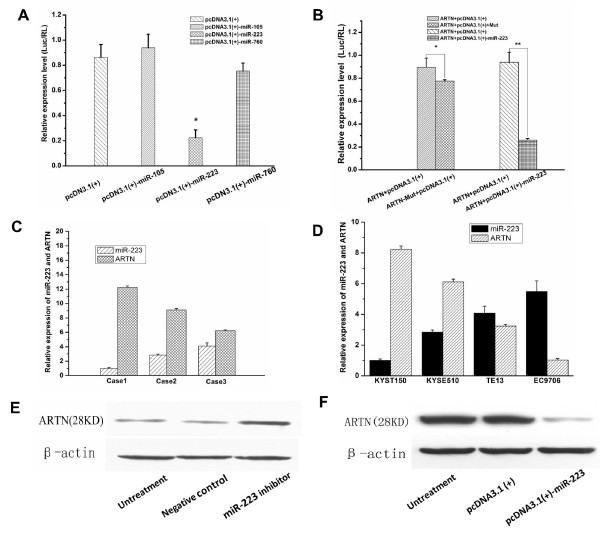
**miR-223 interacts with the ARTN 3'UTR and regulates endogenous ARTN protein expression**. (A) The miR-223 expression vector pcDNA3.1 (+)-miR-223 reduced the activity of firefly luciferase. (B) Mutation of the binding site abolished the ability of miR-223 to inhibit the expression of the luciferase reporter. (C) The relative expression of miR-223 and artemin mRNA in esophageal carcinoma tissues. (D) The relative expression of miR-223 and artemin in esophageal carcinoma cells (KYSE150, KYSE510, TE13 and EC9706). (E) miR-223 inhibitor enhanced the expression of ARTN in EC9706 cells. (F) Overexpression of miR-223 reduced the expression of ARTN in KYSE150 cells. Data are representatives of each group or expressed as mean ± SEM of from three separate experiments.

In order to clarify which miRNA is capable of regulating ARTN protein expression via binding to the 3'UTR of ARTN, we cloned the full length ARTN 3'UTR from a cDNA library downstream of the firefly luciferase coding region in pMIR-GLOTM luciferase vector. We also made mutations to the putative binding site (Additional file 1, Figure S1B). miR-105, miR-223 and miR-760 including a 200 bp flanking sequence were cloned from human genomic DNA and inserted into pcDNA3.1 (+) multiple cloning sites to construct the miRNAs expression vectors. The human embryonic kidney cell line 293 (HEK293) was used for the reporter assay. HEK293 cells were cultured and transfected with pMIR-ARTN and miRNA expression vector or pcDNA3.1 (+) empty vector. After 48 h, the cells were harvested, and the protein was extracted for the luciferase assay. The miR-223 expression vector pcDNA3.1 (+)-miR-223 reduced the firefly luciferase activity (Figure 3A). Subsequently, transfections were carried out in HEK293 cells with pMIR-ARTN and pcDNA3.1(+)-miR-223 or pMIR-ARTN-Mut and pcDNA3.1(+)-miR-223. Mutation of the binding site abolished the ability of miR-223 to inhibit the expression of the luciferase reporter (Figure 3B).

To further study the potential relationship between miR-223 and ARTN, we analyzed miR-223 and ARTN expression level in esophageal cancer tissues. The negative correlation of miR-223 and ARTN expression was found (Figure 3C). We also analyzed miR-223 expression level in four esophageal squamous cell lines (KYSE150, KYSE510, TE13 and EC9706). High expression of miR-223 was detected in KYSE150 and KYST510 cell lines, while low expression of miR-223 was detected in EC9706 and TE13 cell lines (Figure 3D). Interestingly, ARTN expression levels were higher in KYSE150 and KYSE510 than in EC9706 and TE13 cell lines, suggesting that expression of miR-223 was inversely related to ARTN protein level in esophageal carcinoma. KYSE150 and EC9706 were used as models to further investigate the function of miR-223 in esophageal squamous cell carcinoma. We transfected pcDNA3.1 (+)-miR-223 and pcDNA3.1 (+) empty vector into KYSE150 cells and observed a decrease of ARTN protein level in the presence if miR-223 (Figure 3E). Consistent with this result, silencing of miR-223 using an miR-223 inhibitor resulted in an increase of ARTN protein level in EC9706 cells (Figure 3F). These results indicate that ARTN is post-transcriptionally regulated by miR-223.

### Effect of mir-223 on the migration of esophageal carcinoma cells

The MTT assay was used to study cell proliferation. KYSE150 cells were transfected with pcDNA3.1 (+) or pcDNA3.1 (+)-miR-223 prior to the proliferation assy. The overexpression of mir-223 did not inhibit proliferation of KYSE150 cells. The morphology of KYSE150 cells transfected with either pcDNA3.1 (+) or pcDNA3.1 (+)-miR-223 did not change compared to the untreated group, and no difference in cell viability between the three groups was observed (Additional file 2, Figure S2A). In EC9706 cells, the morphology and proliferation did not change when the cells were transfected with a mir-223 inhibitor or Scr-miR-223. (Additional file 2, Figure S2B). The MTT assay demonstrates that miR-223 does not significantly influence the proliferation of esophageal carcinoma cells.

**Figure 4:**
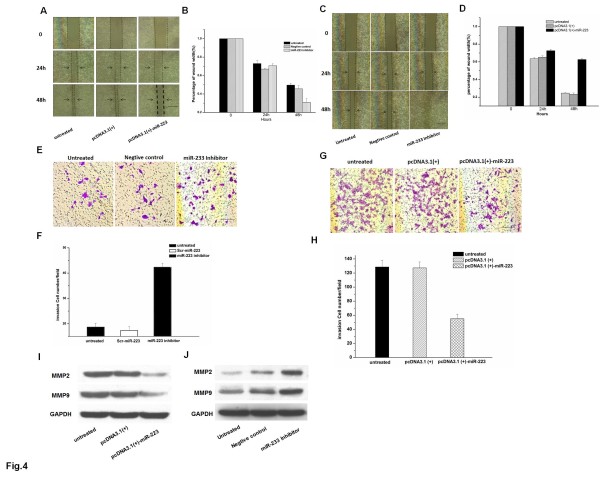
**Effect of miR-223 expression on migration in esophageal carcinoma cells**. (A, B) KYSE150 cells after wounding and during healing. (C, D) The filters were stained with crystal violet and inspected under a microscope (KYSE150 cells). (E, F) The filters were stained with crystal violet and inspected under a microscope (EC9706 cells). (G, H)EC9706 cells after wounding and during healing. (scale bars, 100 μm). (I, J) The expression of mmp2 and mmp9. Scale bars in microscope is 100 μm. Data are representative of each group or expressed as mean ± SEM from three separate experiments.

Scratch-wound healing assays were used to evaluate the effect of miR-223 on cell migration. To explore the potential role of miR-223 in the migration of esophageal carcinoma cell lines, we first examined the effect of the overexpression of miR-223 in KYSE150 cells, which have very low levels of endogenous miR-223, on cell migration. KYSE150 cells transfected with pcDNA3.1 (+)-miR-223 closed the scratch-wounds more slowly than cells that were untreated or transfected with pcDNA3.1 (+) (Figure 4A, B). Meanwhile, EC9706 cells transfected with either an miR-223 inhibitor or Scr-miR-223 closed the scratch-wounds more quickly than cells that were untreated or transfected with pcDNA3.1(+) (Figure 4C, D).

To examine invasion, KYSE150 cells were transfected with either pcDNA3.1 (+)-miR-223 or pcDNA3.1 (+) and reseeded on top of the insert. Consistent with the migration results, overexpression of miR-223 significantly inhibited invasion of KYSE150 cells (Figure 4E, F). EC9706 cells, which have a high level of endogenous miR-223, were transfected with an miR-223 inhibitor or a negative control. The EC9706 cells transfected with the miR-223 inhibitor could effectively penetrate the chamber and travel to the other side of the membrane (Figure 4G, H). Matrix metalloproteinases (MMPs) comprise a family of secreted or membrane-associated zinc-dependent extracellular endopeptidases that enhance invasion and metastases. To determine if increased invasion observed was associated with concomitant change of MMP levels, expression of MMP-2 and MMP-9 were performed. MMP-2 and MMP-9 levels were decreased following up-regulation of miR-223, whereas MMP-2 and MMP-9 levels were increased by inhibition of miR-223 (Figure 4I, G). These observations indicate that miR-223 can inhibit invasion in human esophageal carcinoma cell lines.

## Discussion

Tumor metastasis is a complex multistep process including cell adhesion, migration, angiogenesis, immune escape, and homing to target organs. Cell motility, invasion and invasion are essential features of the metastatic process. Identifying the molecules and pathways that control cell motility and invasion is critical to understanding cancer metastasis. Accumulating evidence is suggesting that ARTN is involved in cancer metastasis. ARTN has been shown to promote cell migration and invasion in human endometrial cell line [8]. In addition, depletion of ARTN can inhibit breast cancer metastasis *in vivo *[5]. Consistent with these results, ARTN overexpression induces cell migration and invasion in pancreatic cancer cells [6,7]. These observations, taken together, indicate that ARTN plays a positive role in migration and invasion of cancer cells. In the present study, we demonstrate that ARTN expression is significantly higher in esophageal carcinoma than in adjacent noninvasive tissues, and that ARTN promotes migration and invasion of esophageal carcinoma cells.

Recently, emerging evidence has implicated miRNAs in metastasis of human cancer. To verify whether ARTN expression is regulated by miRNAs, miR-105, miR-223 and miR-760 were chosen for further study according to the results of a bioinformatic search. We found that miR-223 interacts with the ARTN 3'UTR and regulates endogenous ARTN protein expression. miR-223 was found to be downregulated in breast and ovarian cancer specimens compared to normal ovarian tissues [23]. Down-regulation of miR-223 has *in vivo *significance in chronic lymphocytic leukemia and improves disease risk stratification [19]. In the present study, overexpression of miR-223 leads to a decrease in ARTN function and represses cellular migration and invasion in KYSE150 cells. On the contrary, silencing of miR-223 increases ARTN expression and promotes cellular migration and invasion in EC9706 cells.

## Conclusions

In conclusion, ARTN expression levels were significantly higher in esophageal carcinoma than in adjacent noninvasive tissues, and ARTN promoted migration and invasion of esophageal carcinoma cells. Furthermore, ARTN mRNA was a direct and functional target of miR-223. Finally, we revealed that miR-223 overexpression repressed cell migration and invasion in human esophageal carcinoma cell lines. We provided strong evidence that supports a role for ARTN in tumor cell migration and invasion. Future studies should examine how miR-223 is regulated in human cancer and other human diseases and whether both ARTN and miR-223 are targets for therapy.

## Competing interests

The authors declare that they have no competing interests.

## Authors' contributions

SL, ZL and FG drafted the manuscript, SL, JY, QZ, FG and XQ participated in the design of the study and did most of the experiments, SL, ZL, JY and QZ conceived of the study, BL, and LS participated in its design and coordination, SL, FG, ZS and QZ revised the paper and gave some suggestions. All authors read and approved the final version of the manuscript.

## Supplementary Material

Additional file 1 **Figure S1. The miRNA binding sites in ARTN**. (A) The location of seed sites for miR-105, miR-223 and miR-760 within the ARTN 3'UTR. The three binding sites are highly conserved among species. (B) Construction of the firefly luciferase reporter gene for ARTN. Mutation sites are shown. Click here for file

Additional file 2 **Figure S2. miR-223 has no effect on the proliferation of esophageal carcinoma cells**. (A) Proliferation assay in KYSE150 cells using MTT. (B) Proliferation assay in EC9706 cells. Click here for file
