# Supplementary figures and images for: miR-223 regulates migration and invasion by targeting Artemin in human esophageal carcinoma
Source: J Biomed Sci. 2011 Mar 31;18(1):24. doi: 10.1186/1423-0127-18-24 (PMC3080798; doi:10.1186/1423-0127-18-24)

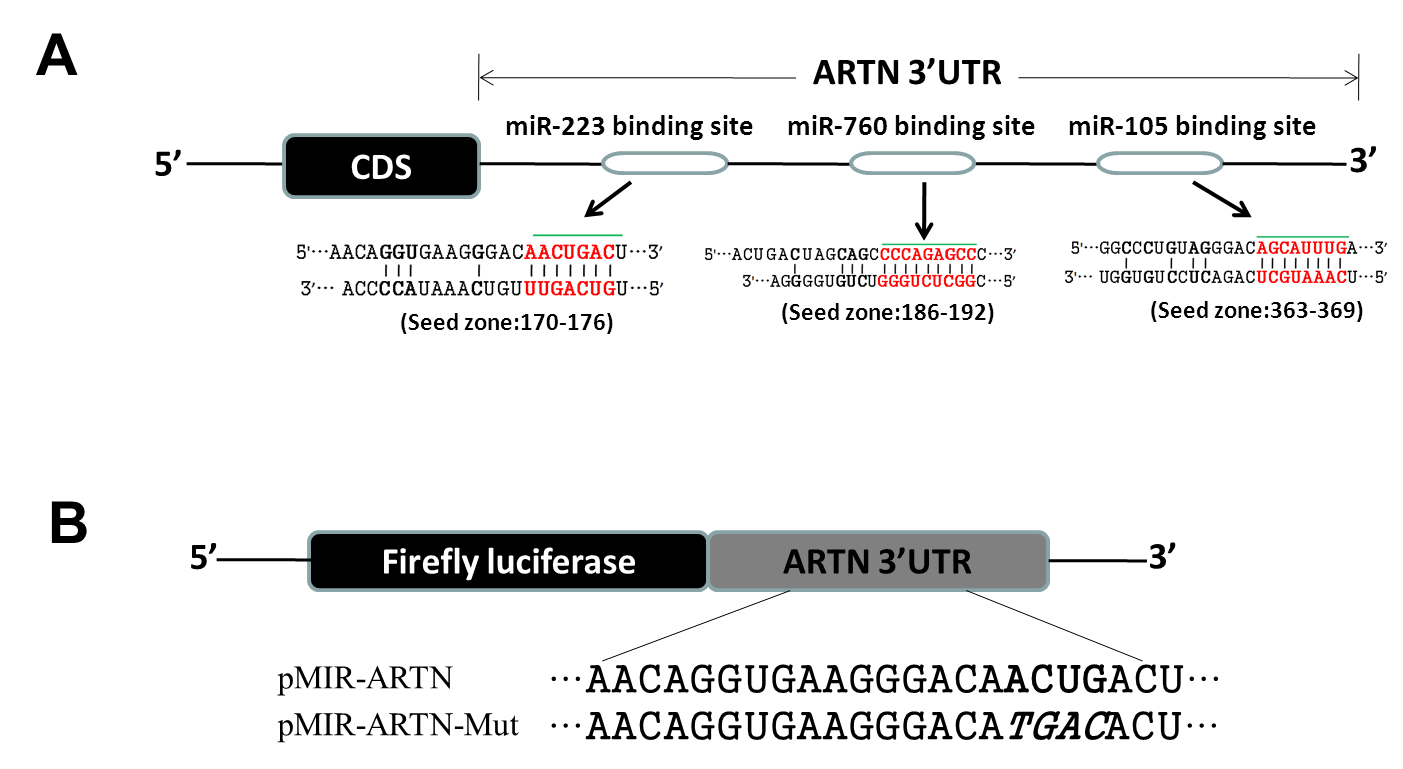

Supplement: Additional file 1 — Figure S1. The miRNA binding sites in ARTN. (A) The location of seed sites for miR-105, miR-223 and miR-760 within the ARTN 3'UTR. The three binding sites are highly conserved among species. (B) Construction of the firefly luciferase reporter gene for ARTN. Mutation sites are shown. [file 1423-0127-18-24-S1.TIFF]

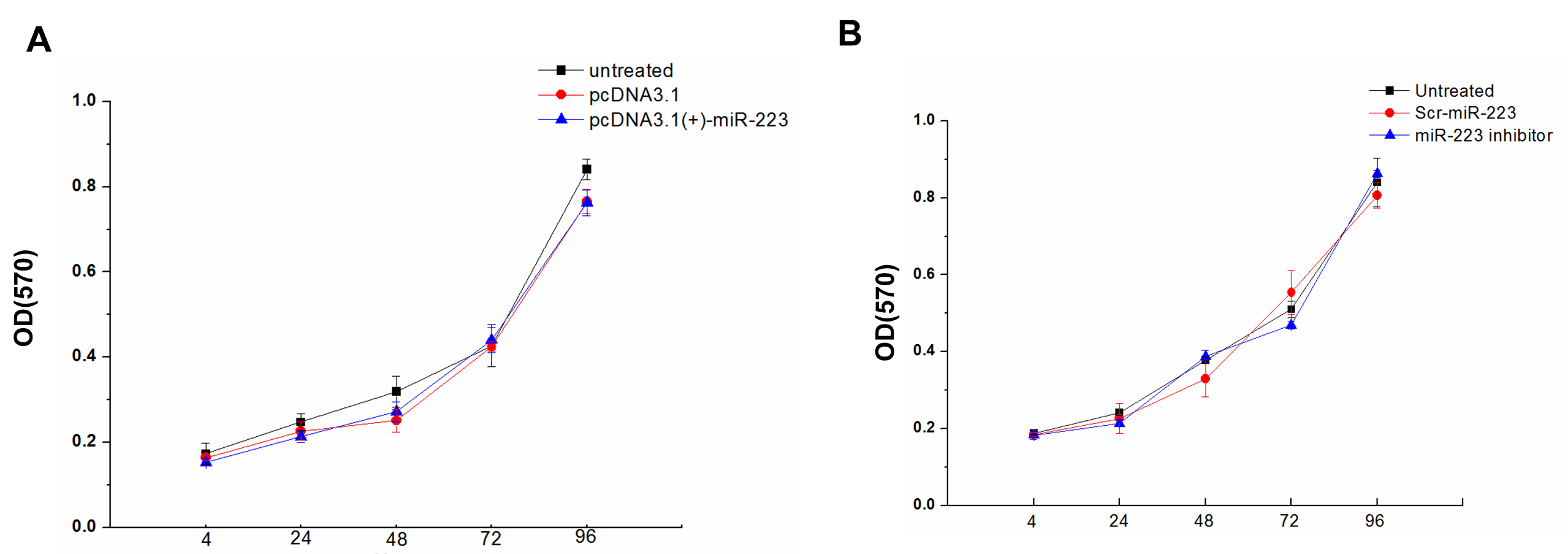

Supplement: Additional file 2 — Figure S2. miR-223 has no effect on the proliferation of esophageal carcinoma cells. (A) Proliferation assay in KYSE150 cells using MTT. (B) Proliferation assay in EC9706 cells. [file 1423-0127-18-24-S2.TIFF]
